# *Hieracium sinoaestivum* (Asteraceae), a new species from North China

**DOI:** 10.3897/phytokeys.39.7788

**Published:** 2014-06-20

**Authors:** Alexander N. Sennikov

**Affiliations:** 1 Botanical Museum, Finnish Museum of Natural History, P.O. Box 7, 00014 University of Helsinki, Finland; 2 Herbarium, Komarov Botanical Institute of Russian Academy of Sciences, Prof. Popov str. 2, 197376 St. Petersburg, Russia

**Keywords:** Apomictic species, boreal forest, Compositae, Shanxi, Siberia, taiga

## Abstract

*Hieracium sinoaestivum* Sennikov **sp. nov.** is described as new to science and illustrated. This presumably apomictic species is solely known from two old collections made in a single locality in the Shanxi Province of China. It belongs to the hybridogenous group *Hieracium* sect. *Aestiva* (*Hieracium* sect. *Prenanthoidea* × *Hieracium* sect. *Umbellata*) and is most similar to *Hieracium veresczaginii* from southern Siberia. The new species occurs at low altitudes in the forest belt of Lülian Mts. and belongs to taiga forest elements.

## Introduction

The genus *Hieracium* L. with its ca. 10000 species (Sell and Murrell 2006), the majority of which are presumably apomictic (Chrtek et al. 2007), has the greatest diversity in the mountains of Europe and the Caucasus (Zahn 1921–1922). In China the genus is on the very margin of its distribution and is represented by a few taxonomic groups and species, mostly found in the mountains of Central Asia (Sennikov 2008, Shih and Gottschlich 2011).

In spite of the very low number of species, the genus is still quite poorly studied in China. The latest authoritative sources give different statistics. The Chinese edition of the *Flora of China* (Shih 1997) accepted four species which are referable to the present-day *Hieracium* (excluding *Pilosella* Vaill., *Hololeion* Kitam. and one misplaced species of *Crepis*). A revised treatment of *Hieracium* in Central Asia (Sennikov 2008) included eight species, of which four (*Hieracium kirghizorum* Üksip, *Hieracium krylovii* Nevski ex Schljakov, *Hieracium robustum* Fr., *Hieracium subramosum* Lönnr.) were new to China. The English edition of the *Flora of China* (Shih and Gottschlich 2011) revised the old treatment, recognizing *Hieracium robustum* and *Hieracium morii* Hayata from Taiwan and adding *Crepis shawanensis* C. Shih to the synonymy of *Hieracium korshinskyi* Zahn, but they kept the number of species low. Shih and Gottschlich’s treatment accepted only six species, probably because the authors had little access to the material from Central Asia.

During my revision of assorted *Hieracium* specimens collected in Asia and kept in the Swedish Museum of Natural History, Stockholm (S), I recovered two gatherings of a plant which was recognised as a new species many years ago by the prominent Swedish *Hieracium* expert Karl Johansson (1856–1928). Johansson compiled a detailed species description that was attached to one of the specimens, both handwritten and in typescript, and it was obviously his death that prevented him from publication of this novelty.

This species is highly dissimilar from any species of *Hieracium* hitherto known from China, and therefore is here described as new to science. The species name suggested by Johansson, “*Hieracium chinense* Johanss.”, may not be used because of the earlier near-homonym *Hieracium sinense* Vaniot (1903); the use of such near-homonyms is explicitly precluded by Art. 53.3 with Ex. 11 (McNeill et al. 2012).

## Materials and methods

The new species was described solely on the basis of two dried collections kept at S. Measurements were taken with a light microscope (Leica S4E). The species description follows Sennikov (2002) and Sell and Murrell (2006) with minor modifications. Terminology in the descriptions of pubescence follows Schljakov (1989).

Details of pubescence were photographed with a digital camera (Canon EOS 5D Mark III, lens EF 100 mm 1:2.8L, two extension rings), and the series of images was processed with the Helicon Focus Pro software.

The distribution map was produced using the R software environment for statistical computing and graphics (R Development Core Team 2013). The basemap was compiled from the Digital Chart of the World, Arc/INFO resource provided by the Environmental Systems Research Institute, Inc., the Pennsylvania State University Libraries.

## Taxonomic treatment

### Hieracium sinoaestivum

Taxon classificationPlantaeAsteralesAsteraceae

Sennikov sp. nov.

urn:lsid:ipni.org:names:77140258-1

#### Type.

**Figure 1.**
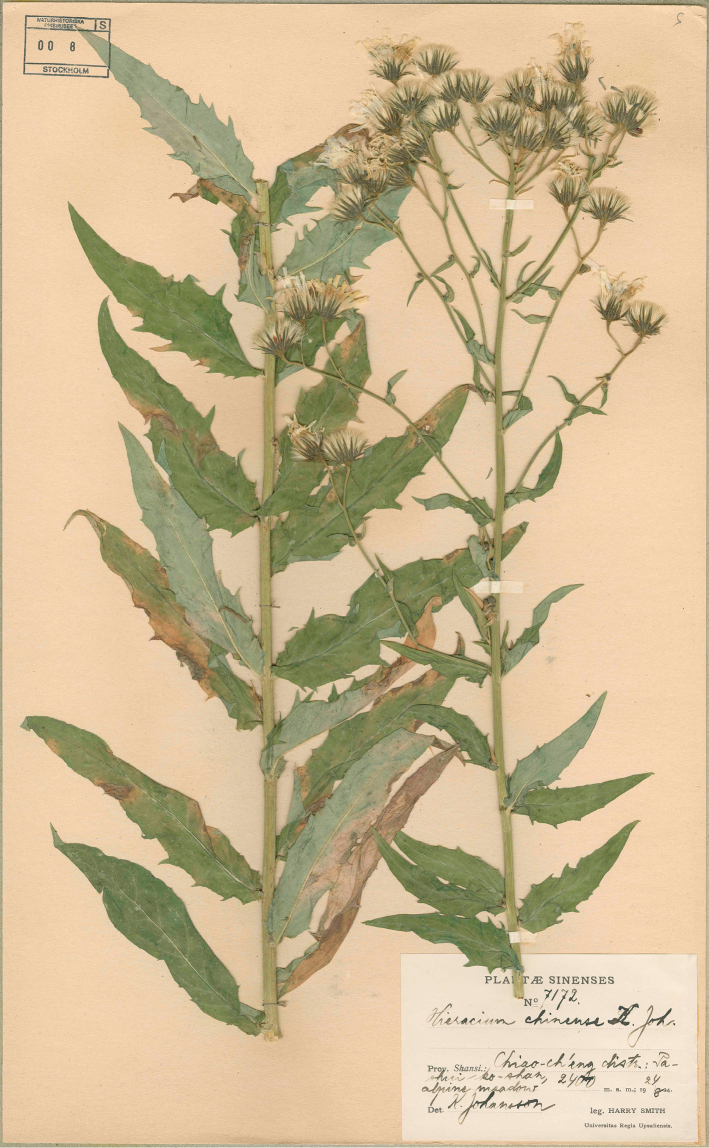
Holotype of *Hieracium sinoaestivum* Sennikov.

China. Shanxi: Lüliang City. “Chiao-ch’eng distr., Pashui-ko-shan”, alpine meadow, 2400 ft., 24.08.1924, *Harry Smith 7172* (S!, holotype; UPS, isotype). Fig. 1.

#### Paratypes.

China. Shanxi: Lüliang City. “Chiao-ch’eng distr., Pashui-ko-shan”, meadows in mixed forests, 2100 ft., 28.08.1924, *Harry Smith 7219* (BM 000996241 photo!, S!).

#### Diagnosis.

The new species differs from the most similar *Hieracium veresczaginii* Schischk. & Serg. mainly in a greater density of simple hairs (rare to sparse vs. solitary or sometimes absent) on the phyllaries.

#### Description.

**Figure 2.**
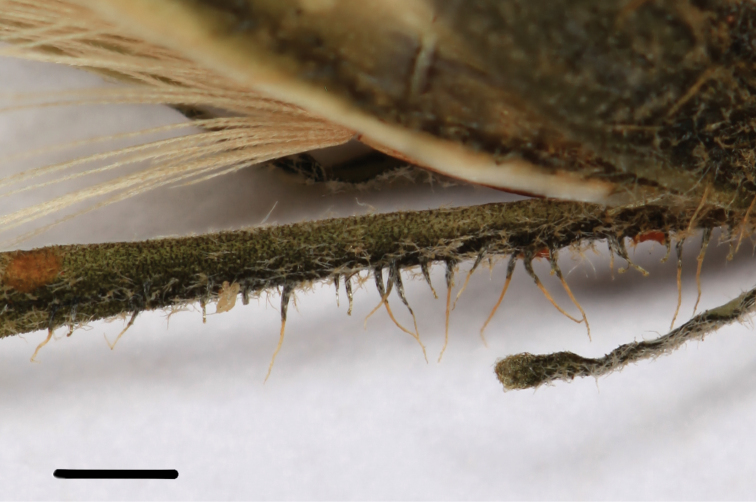
Pubescence on the phyllaries of *Hieracium sinoaestivum* Sennikov (*Harry Smith 7219*, S). Scale bar: 1 mm.

Evidently aphyllopodous *perennial plant*. *Stem* 60–70 cm tall, robust, pale green, without simple hairs (paratype) or with abundant simple hairs up to 3 mm long (holotype), with lax stellate pubescence mostly in the lower half. *Leaves* up to 50, gradually decreasing in size upwards, sessile, clearly bicolour, intensely green on upper surface, pale green beneath, with lax stellate pubescence on both sides and simple hairs 1.5–2 mm long along margins and beneath; the *lower* unknown (withered at anthesis); the lamina of the *median* leaves (most developed) 9–12 cm long, 2.5–4 cm wide (ratio 1:3–3.5), oblong-ovate, widest near basal third, acute at apex, broadly cuneate or rotund at base, with 4–5 pairs of narrow acute teeth up to 5(8) mm long; the lamina of the *upper* leaves up to 6 cm long, 1.5–1.8 cm wide, ovate-lanceolate, widest near base, acute at apex, rotund at base, with 3–4 pairs of small narrow teeth. *Synflorescence* up to 25 cm long, laxly branched with 3–8 branches and 10–35 capitula; branches elongated, without simple and glandular hairs under the capitula, with dense stellate pubescence. *Capitula* cup-shaped, rounded at base. *Phyllaries* (Fig. 2) 9–10 mm long, 1–1.2 mm wide at middle, 1.5–1.7 mm wide at base, oblong-triangular with a gradually narrowed apex, olive green, the inner with paler margins, with simple and glandular hairs along a narrow median line and with stellate hairs over the surface; the inner with rare to sparse (5–15) simple hairs 1–1.2 mm long, dark at base, otherwise whitish, with sparse (up to 20) glandular hairs 0.2–0.5 mm long, thin and rather dark, with lax stellate pubescence, tipped with a few very short cilia at apex. *Florets* 15–18, 16–17 mm long. *Ligules* probably intensely yellow, glabrous-tipped. *Styles* with black spines. *Achenes* ca 4 mm long, brick red. *Pappus* 7–8 mm long, yellowish.

#### Affinity.

The new species is attributed to *Hieracium* sect. *Aestiva* (Üksip ex Schljakov) Sennikov which was circumscribed to embrace morphotypes presumably originated from crosses between members of *Hieracium* sect. *Prenanthoidea* W.D.J.Koch s.l. and *Hieracium* sect. *Umbellata* Sendtn. (Sennikov 1999). *Hieracium sinoaestivum* shares the abundant stellate pubescence, habit and largely the shape of leaves with some broad-leaved forms of *Hieracium umbellatum* L. but differs from the latter in its broader phyllaries with straight (vs. reflexed) tips, its leaf base clearly subrotund (vs. broadly cuneate), and a much greater number of simple and glandular hairs on the phyllaries (the phyllaries in *Hieracium umbellatum* may occasionally have solitary to rare glandular hairs only). From *Hieracium* sect. *Prenanthoidea* the new species borrows a denser indumentum of phyllaries, a broader base of leaves, and the pale green (nearly glaucous) colour of leaves which is untypical of *Hieracium umbellatum*.

Of the presumed parents, *Hieracium umbellatum* is a common component of the boreal vegetation in the mountains of northern and western China (Shih and Gottschlich 2011). In China, the species of *Hieracium* sect. *Prenanthoidea* s.l. (including hybrids) occur in the Xinjiang Province but not in the northern provinces (Sennikov 2008, 2012, Shih and Gottschlich 2011).

No similar species is known from China (Sennikov 2008, Shih and Gottschlich 2011). In southern Siberia *Hieracium* sect. *Aestiva* is represented by about 7 species (Tupitzina 2004), of which only *Hieracium nasimovae* Stepanov and *Hieracium veresczaginii* Schischk. & Serg. are said to have the leaf base cuneate or rotund and the synflorescence branches usually without glandular hairs. Unlike *Hieracium sinoaestivum*, *Hieracium nasimovae* is characterized by a large number of glandular hairs (up to 60) on the phyllaries and by the slightly panduriform leaves (Stepanov 1998); this poorly known local taxon may actually be closely related to *Hieracium krylovii* Nevsky ex Schljakov, a species of *Hieracium* sect. *Aestiva* with a greater expression of characters of *Hieracium* sect. *Prenanthoidea* s.l.

*Hieracium veresczaginii* occurs in eastern Kazakhstan on the border with Russia (Kotukhov 1971, Abdulina 1999) and in southern Siberia westwards to the Chita town (Tupitzina 2004). It is said to be characterized by the phyllaries with sparse glandular hairs 0.2–0.4 mm long (along a median line) and sometimes also with solitary short simple hairs, usually with an abundant stellate pubescence (Tupitzina 2004). My examination of the material of *Hieracium veresczaginii* kept in LE has shown that this species regularly has ovate-lanceolate or oblong-ovate leaves with a rotund base and a coarse dentation, resembling those of large individuals of *Hieracium umbellatum*. In the shape of leaves and the type of pubescence *Hieracium veresczaginii* seems to be the most similar to *Hieracium sinoaestivum*, mainly differing in solitary simple hairs on its phyllaries. It is a species of taiga forest, occurring in spruce, fir, pine, birch and mixed forests of the Altai-Sayan mountain system and its northern extensions (Tupitzina 2004).

#### Variability.

The two original collections clearly differ from each other in the pubescence of stems and leaves, although the indumentum of phyllaries is nearly invariable. The robust and hairy plant of *Harry Smith 7172* also has a much longer dentation of leaves. This difference is considered taxonomically insignificant but likely indicates genetic variability within this presumably apomictic species.

#### Distribution.

**Figure 3.**
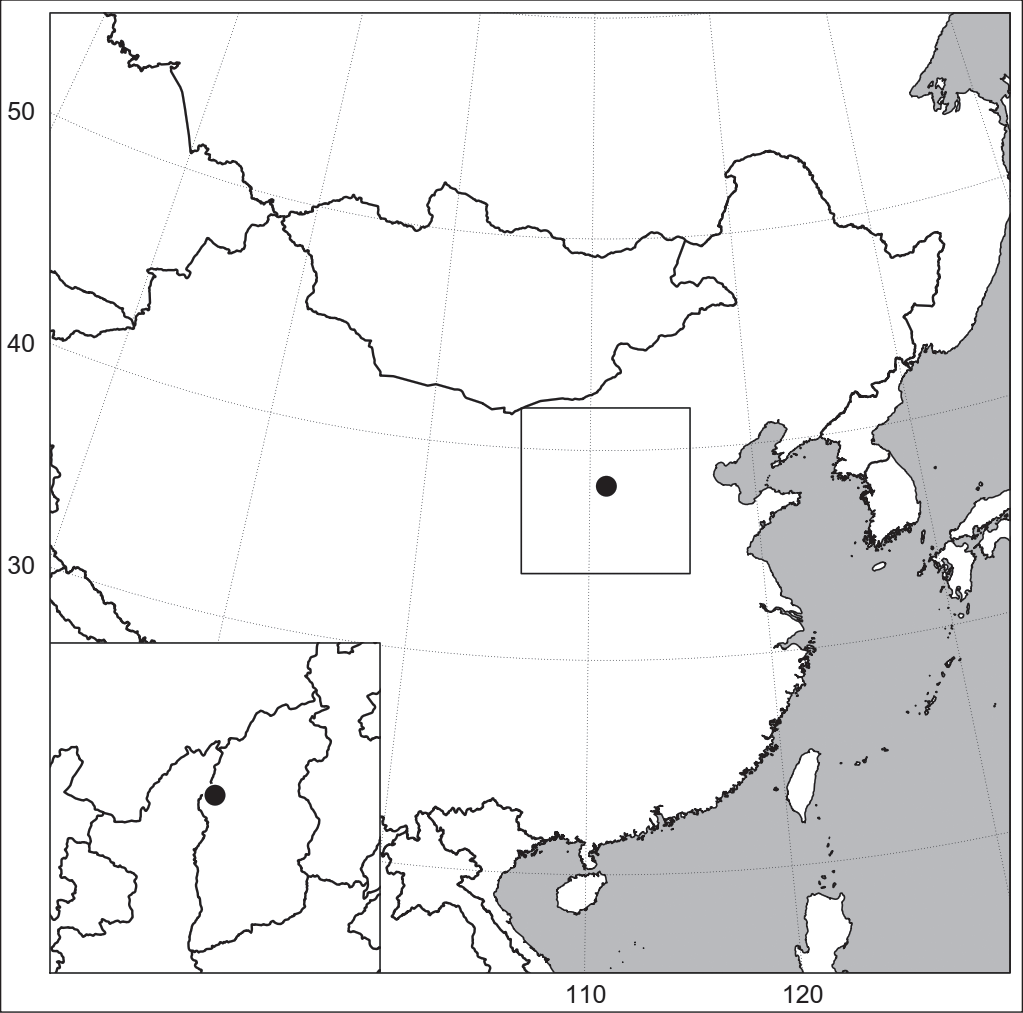
Distribution of *Hieracium sinoaestivum* Sennikov.

The new species is known from a single locality in the Shanxi Province of China, situated approximately at 38.3°N, 111°E in the Lüliang Mountains (Fig. 3).

This locality lies within the distribution area of *Hieracium umbellatum* (Shih and Gottschlich 2011) but at the distance of ca. 600 km from the nearest locality of *Hieracium* sect. *Prenanthoidea* s.l. (including hybrids) in southern Siberia (Tupitzina 2004).

#### Ecology.

According to the collector’s notes, *Hieracium sinoaestivum* grows on montane meadows in the forest belt at altitudes of 600–750 m a.s.l. The species flowers in August, fruits in August–September.

#### Phytogeography.

The only locality of the new species is situated in the subregion of North China Mountains, region of North China, subkingdom of Sino-Japanese Forest, Eastern Asiatic kingdom of Chinese phytogeographers (Sun 2013). This area has a rich indigenous flora, with ca. 300 species endemic to the subregion (Wang 1997, Sun 2013). *Hieracium sinoaestivum* belongs to taiga forest floristic elements and represents a penetration of holarctic elements into the East Asian flora.

#### Conservation status.

Data deficient.

#### Mode of reproduction.

Not known, presumably apomictic.

#### Etymology.

The species epithet is derived from *Sino*-, pertaining to China, and *aestivum*, reflecting the sectional placement of the species.

## Supplementary Material

XML Treatment for Hieracium sinoaestivum
